# Supplementary material for: Wide Concentration Range of Tb3+ Doping Influence on Scintillation Properties of (Ce, Tb, Gd)3Ga2Al3O12 Crystals Grown by the Optical Floating Zone Method
Source: Materials (Basel). 2022 Mar 10;15(6):2044. doi: 10.3390/ma15062044 (PMC8951279; doi:10.3390/ma15062044)
Supplement: Supplementary file 1 [file materials-15-02044-s001.zip › materials-1557670-supplementary.pdf]

materials

MDPI

Supplementary Materials

Wide Concentration Range of Tb^{3+} Doping Influence on Scintillation Properties of (Ce, Tb, Gd)$_3$Ga$_2$Al$_3$O$_{12}$ Crystals Grown by the Optical Floating Zone Method

Tong Wu [1,2], Ling Wang [1,2], Yun Shi [1,3,*], Xintang Huang [2,*], Qian Zhang [1,4], Yifei Xiong [1,5], Hui Wang [1], Jinghong Fang [1], Jinqi Ni [1], Huan He [1], Chaoyue Wang [1], Zhenzhen Zhou [1], Qian Liu [1], Qin Li [1], Jianding Yu [1,3], Oleg Shichalin [6] and Evgeniy Papynov [6]

1 State Key Laboratory of High Performance Ceramics and Superfine Microstructure, Shanghai Institute of Ceramics, Chinese Academy of Sciences, Shanghai 200050, China; wutong1@mails.ccnu.edu.cn (T.W.); wll1327201@outlook.com (L.W.); zq1421849356@163.com (Q.Z.); xiongyifei1996@163.com (Y.X.); wanghui@mail.sic.ac.cn (H.W.); fangjinghong@mail.sic.ac.cn (J.F.); nijinqi@mail.sic.ac.cn (J.N.); ch.hh@mail.sic.ac.cn (H.H.); cywang@mail.sic.ac.cn (C.W.); zhouzhenzhen@mail.sic.ac.cn (Z.Z.); qianliu@mail.sic.ac.cn (Q.L.); liqin@mail.sic.ac.cn (Q.L.); yujianding@mail.sic.ac.cn (J.Y.);

2 College of Physical Science and Technology, Central China Normal University, Wuhan 430079, China

3 Center of Materials Science and Optoelectronics Engineering, University of Chinese Academy of Sciences, Beijing 100049, China

4 School of Materials Science and Engineering, Zhengzhou University, Zhengzhou 450001, China;

5 College of Material Science and Engineering, Nanjing Tech University, Nanjing 211816, China

6 Laboratory of nuclear technology, Institute of High Technologies and Advanced Materials, Far Eastern Federal University, Vladivostok 690091, Russia oleg_shich@mail.ru (O.S.); papynov@mail.ru (E.P.)

* Correspondence: shiyun@mail.sic.ac.cn (Y.S.); xthuang@mail.ccnu.edu.cn (X.H.)

Citation: Wu, T.; Wang, L.; Shi, Y.; Huang, X.; Zhang, Q.; Xiong, Y.; Wang, H.; Fang, J.; Ni, J.; He, H.; et al. Wide Concentration Range of Tb^{3+} Doping Influence on Scintillation Properties of (Ce, Tb, Gd)$_3$Ga$_2$Al$_3$O$_{12}$ Crystals Grown by the Optical Floating Zone Method. *Materials* **2022**, *15*, 2044. https://doi.org/10.3390/ma15062044

Academic Editors: Alexander A. Lebedev and Giorgio Biasiol

Received: 30 December 2021
Accepted: 4 March 2022
Published: 10 March 2022

Publisher's Note: MDPI stays neutral with regard to jurisdictional claims in published maps and institutional affiliations.

Copyright: © 2022 by the authors. Licensee MDPI, Basel, Switzerland. This article is an open access article distributed under the terms and conditions of the Creative Commons Attribution (CC BY) license (https://creativecommons.org/licenses/by/4.0/).

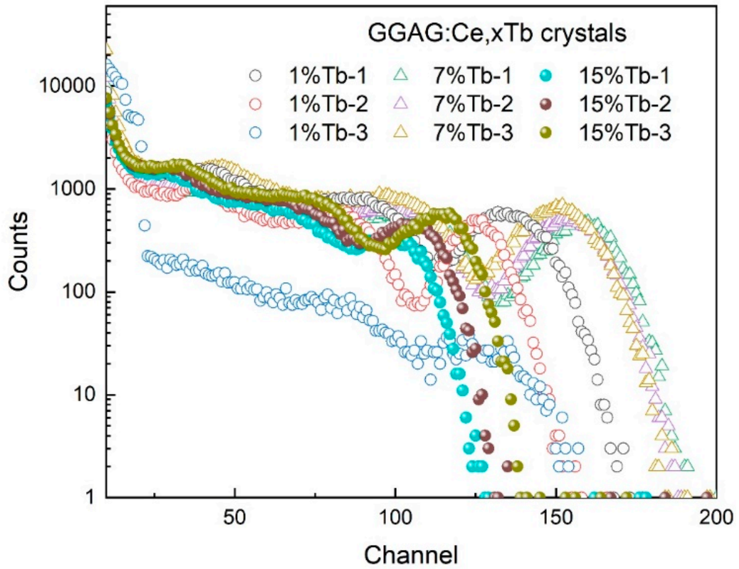

Figure S1. Pulse height spectra of the as grown GGAG:Ce, xTb (x = 1, 7, 15 at.%) crystals. The shaping time is 0.75 μs, under gamma ray (^{137}Cs 662 keV source) (1, 2 and 3 represent three crystals of the same component at different locations).

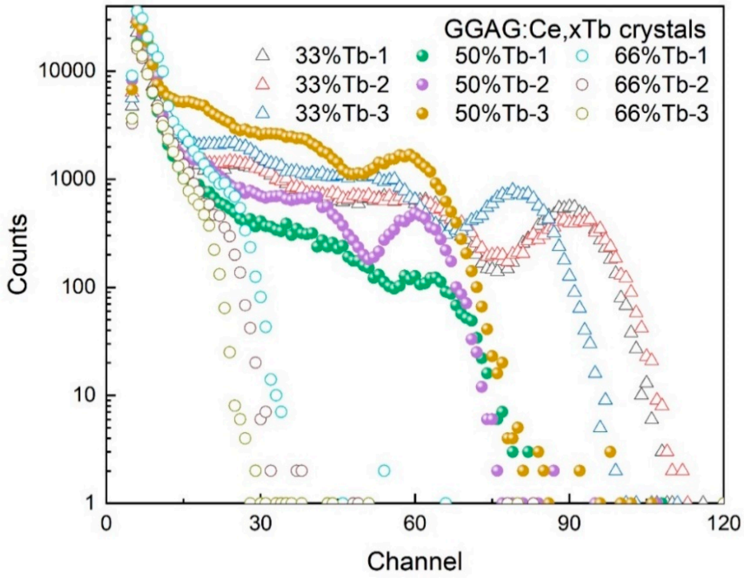

Figure S2. Pulse height spectra of the as grown GGAG:Ce, xTb (x = 33, 50, 66 at.%) crystals. The shaping time is 0.75 μs, under gamma ray (^{137}Cs 662 keV source) (1, 2 and 3 represent three crystals of the same component at different locations).

Table S1. Calculated Relative Light Yield with respect to the standard GGAG:Ce crystal (channel 481.6) of the as grown GGAG:Ce, xTb (x = 1, 7, 15, 33, 50, 66 at.%) crystals under gamma ray excitation (^{137}Cs 662 keV source).

Samples	Channel Number (Light Yield/pho·MeV-1)			
	1	2	3	4
GGAG:Ce, 1%Tb	134.27 (16170)	124.46 (14989)	133.76 (16109)	—
GGAG:Ce, 7%Tb	157.28 (18942)	152.69 (18389)	149.13 (17960)	—
GGAG:Ce, 15%Tb	100.03 (12046)	105.11 (12659)	113.78 (13703)	—
GGAG:Ce, 33%Tb	88.46 (10653)	90.38 (10885)	79.02 (9517)	91.39 (11006)
GGAG:Ce, 50%Tb	61.32 (7385)	59.75 (7196)	57.79 (6960)	55.03 (6627)
GGAG:Ce, 66%Tb	23 (2770)	20 (2409)	18 (2168)	30.84 (3714)
